# Representations of reproductive difficulties in women participating in support groups

**DOI:** 10.1192/j.eurpsy.2022.2207

**Published:** 2022-09-01

**Authors:** E. Bityutskaya, E. Vorontsova, N. Lebedeva

**Affiliations:** 1 Lomonosov Moscow State University, Faculty Of Psychology, Moscow, Russian Federation; 2 Non-profit organization for women with reproductive issues «You are not alone», Research Department, Moscow, Russian Federation; 3 Moscow Metropolitan Governance University, Diagnostics Department, Moscow, Russian Federation

**Keywords:** approach coping, avoidance coping, representation of difficulties

## Abstract

**Introduction:**

Women with reproductive difficulties often feel stigmatized and isolated. Information concerning their specific experience can help plan psychosocial interventions.

**Objectives:**

The study aims to analyze reproductive difficulties representations in women with different coping orientations.

**Methods:**

Participants: 48 women (aged 24-43) from support groups arranged by the “You Are Not Alone” non-profit organization for women with reproductive issues. Based on the questionnaire “Types of Orientations in Difficult Situation”, participants were divided into three groups: approach coping (N=16), avoidance coping (N=9), ambivalent coping (N=23). Content analysis was conducted based on stories about reproduction difficulties experiences.

**Results:**

**Table 1:** 

	Groups		
Categories	Approach coping	Ambivalent coping	Avoidance coping
**1.Emotions**			
Negative emotions	31%	43%	
Hope	31%		
Mobilization to solve the problem		48%	
Severe emotional state		9%	67%
No emotion	38%		33%
**2.Goals**			
Birth of a child	81%	61%	44%
Acceptance/inner harmony	13%	26%	
Understanding the cause of difficulties	7%	13%	
Maintaining the integrity of the body			56%
**3.Worst-case scenario**			
No child	56%	70%	33%
Illness/depression/insanity		13%	44%
Own death	13%	4%	22%
Denial of the possibility of the worst-case scenario	19%	9%	
**4.Best-case scenario**			
Having a child	100%	91%	67%
Accepting infertility		6%	
Improving own health			33%
**Objective indicator:** perinatal losses	6%	27%	14%

**Conclusions:**

We identified three types of representation of reproductive difficulties in women: approaching the goal of having a child; avoidance (fear of own death/illness/insanity or not having a child); ambivalent coping (alternating approach/avoidance). Funding: The study was funded by RFBR, project number 20-013-00838.

**Disclosure:**

No significant relationships.

